# An *in vitro* Study of Diffusibility and Degradation of Three Calcium Hydroxide Pastes

**DOI:** 10.5005/jp-journals-10005-1075

**Published:** 2011-04-15

**Authors:** Shailendra Gupta

**Affiliations:** Professor and Head, Department of Conservative Dentistry and Endodontics, Mahatma Gandhi Dental College and Hospital Sitapura, Jaipur, Rajasthan, India

**Keywords:** Calcium hydroxide, CMCP, Propylene glycol, Mordant black indicator.

## Abstract

**Aims and objectives :** The aim of this *in vitro* study is to assess the diffusibility and degradation of three calcium hydroxide pastes.

**Materials and methods :** Three pastes were prepared by mixing calcium hydroxide powder with three different vehicles namely water, propylene glycol and CMCP for the investigation. The three pastes were sealed in porcelain caps and immersed in distilled water for the diffusion study. The change in the pH of the surrounding water indicated the rate and degree of diffusion in the degradation study (1 to 7 days, 14th day, 21st day and 30th day). The pastes were exposed to an atmosphere of carbon dioxide in the degradation study to assess the degree to which they can undergo degradation by carbonation. Mordant Black or Solochrome Black indicator was used for the estimation.

**Results and conclusion :** Calcium hydroxide readily diffuses from all the three pastes. Calcium hydroxide diffuses marginally less from a paste made with propylene glycol as compared to CMCP paste. The comparative stability of Ca(OH)_2_ was studied and the results showed that consistently significant protection is imparted to Ca(OH)_2_ by CMCP over widely ranging periods.

## INTRODUCTION

Endodontic intervention has expanded the scope of dentistry by retaining teeth which were previously destined for extraction. Over the years, the rationale has been always on the change. The scope of endodontics is to render the affected tooth biologically acceptable. Symptom free, functional and without any diagnosable pathosis.

Endodontic triad consists of cleaning, sterilizing (sanitizing) and obturating the root canal. Bacteria may effectively be controlled or eliminated from diseased canals by two means:

1. Debridement and proper irrigation while cleaning and shaping the canal.
2. Intracanal medication. The rationale for medicating the pulp cavity was that the root canal had to be disinfected, prior to its preparation or following it, before final obturation.^1,2^

Calcium hydroxide was introduced by Herman (1930) and appears to be a single medicament which has been proved to perform several of the functions expected of an ideal root canal medicament, and its use as a paste is widely accepted. The various vehicles recommended with calcium hydroxide are distilled water, normal saline, methyl cellulose, anesthetic solution, metacresyl acetate, chlorothymol, Ringer solution, propylene glycol and CMCP.^3-5^

Calcium hydroxide is used in variety of clinical situations ranging from indirect pulp capping to conservative management of large apical pathologies and promoting apexification. It is accredited with antimicrobial activity, as it can impart high alkalinity of the order of 12 pH. Its high bactericidal effect, biocompatibility, ability to induce local calcific response, efficacy in controlling resorptions and to control exudates in root canals are well-documented.

Calcium hydroxide preparations has been subjected to extensive laboratory and clinical studies. It is a known fact that calcium hydroxide can undergo chemical changes relatively easily when exposed to unfavorable environmental conditions. Its biological qualities can be adversely effected by the degradation. The two important aspects of its qualities that require further serious scrutiny are diffusibility and degradation, it undergoes while in use, as these have a direct influence on its therapeutic value.

Hence, the main aim of the study is to assess/study *in vitro,* the diffusibility and degradation of calcium hydroxide from pastes prepared by using different vehicles.

## MATERIALS AND METHODS

### Diffusibility of Ca(OH)_2_ Pastes

The present investigation is an *in vitro* study to determine the diffusibility of calcium hydroxide pastes using propylene glycol 100%, camphorated parachlorophenol (CMCP) and distilled water. For this study, three materials were used in combination with calcium hydroxide, which are CMCP, distilled water and propylene glycol.

Materials Used for this Study

1. Calcium hydroxide powder (Laboratory reagent, Chemical Division, Glaxo Laboratories India Ltd, Mumbai).
2. 100% propylene glycol liquid (Analytic reagent, SD Fine Chem Pvt Ltd, Boisar).
3. Camphorated parachlorophenol liquid prepared freshly by mixing in a water bath:
  –  65 parts of Camphor (Micro Analytic Reagent Laboratory Chemicals, Mumbai).
  –  Plus 35 parts of Parachlorophenol (Zur Synthese, Matk-Schuchardt).
4. Distilled water:
  –  Ca(OH)_2_ + CMCP
  –  Ca(OH)_2_ + propylene glycol 100%
  –  Ca(OH)_2_ + distilled water.

All the three Ca(OH)_2_ pastes were made by mixing 2 gm of Ca(OH)_2_ with 3 ml of the appropriate liquid on the glass slab. Calcium hydroxide Ca(OH)_2_ powder was preweighed and then stored in autoclaved vials.

**Fig. 1:**
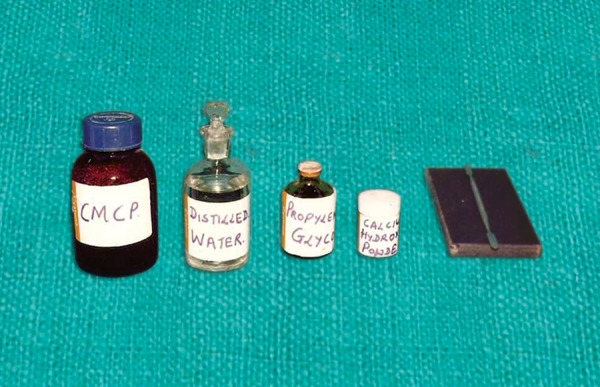
Amboured colored bottle containing CMCP; Distilled water; Propylene glycol and calcium hydroxide with glass slab and spatula

Armamentarium (Fig. 1)

- Glass slab
- Cement spatula
- Eighteen wide mouthed bottles with caps
- pH paper with a range of 6.5 to 9.0 (Glaxo Laboratories India Ltd, Mumbai)
- Porcelain caps (18 in number)
- Modelling wax (Hindustan Dental Products Ltd, Mumbai)

Separate instruments (like glass slab, cement spatula) were used for the preparation of each paste under sterile conditions.

Method

The technique used for this investigation was to determine the rate of diffusion of Ca(OH)_2_ from various formulations that have been studied.

Porcelain caps (18 in number) were sealed from one end with modeling wax. Calcium hydroxide powder was taken on a glass slab, i.e. 2 gm with 3 ml of the liquid, either CMCP or propylene glycol or distilled water was mixed and inserted from one side (from the open and into the porcelain caps). All the 18 porcelain caps were sealed with fast setting glue called Elsic and left for 15 to 20 minutes to dry.

Each of the eighteen wide mouthed bottles was filled with 40 ml of distilled water. Each specimen was kept in the wide mouthed bottles and closed. Six specimens were taken containing porcelain caps filled with CMCP, propylene glycol or distilled water.

**Fig. 2:**
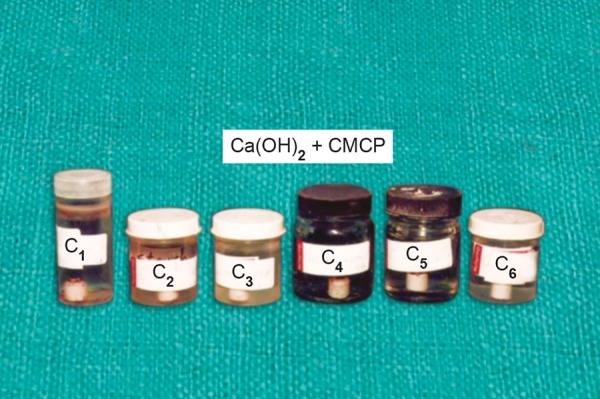
Bottles containing 40 ml of distilled water with porcelain caps immersed into it having a paste of Ca (OH)_2_ + CMCP

**Fig. 3:**
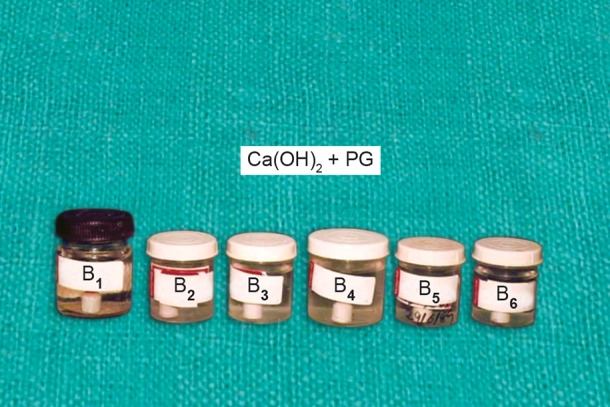
Six bottles containing 40 ml of distilled water with porcelain caps immersed into it having a paste of a Ca(OH)_2_ + PG

**Fig. 4:**
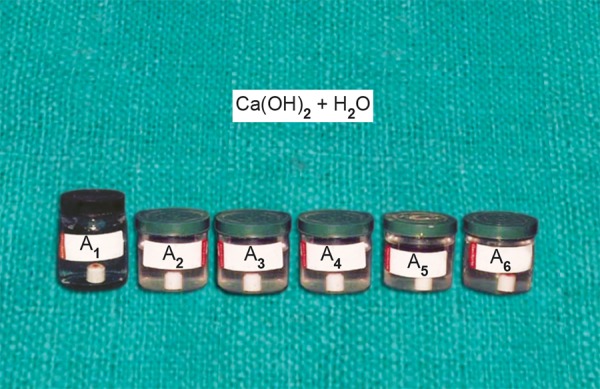
Six bottles containing 40 ml of distilled water with porcelain caps immersed into it having a paste of a Ca(OH)_2_ + H_2_O

- Ca(OH)_2_ + CMCP was marked as C_1_, C_2_, C_3_, C_4_, C_5_ and C_6_ (Fig. 2)
- Ca(OH)_2_ + propylene glycol was marked as B_1_, B_2_, B_3_, B_4_, B_5_ and B_6_ (Fig. 3)
- Ca(OH)_2_ + distilled waster was marked as A_1_, A_2_, A_3_, A_4_, A_5_ and A_6_ (Fig. 4).

pH was noted down from 1st, 2nd, 4th, 5 th, 6th and 7th day. Later on the pH was noted down on 14th, 21st and on 30th day.

pH of these specimens were noted down after the required period. The porcelain caps were inverted and kept immersed in 40 ml of distilled water in wide mouthed bottles.

### Rate of CO_2_ Absorption by Calcium Hydroxide Powder

The present investigation is an *in vitro* study to determine the rate of CO_2_ absorption by calcium hydroxide powder using propylene glycol (100%), camphorated parachlorophenol (CMCP) and distilled water. For this study, three materials were used in combination with calcium hydroxide and they are: CMCP, propylene glycol and distilled water.

Materials used for this Study

1. Calcium hydroxide powder (Laboratory reagent, Chemical Division, Glaxo Laboratories India Ltd, Mumbai).
2. 100% propylene glycol liquid (Analytic reagent, SD Fine Chem Pvt Ltd, Boisar).
3. Camphorated parachlorophenol liquid prepared freshly by mixing in a water bath:
  - 65 parts of Camphor (Micro Analytic Reagent, Labo Chemicals, Mumbai).
  - Plus 35 parts of Parachlorophenol (Zur Synthese, Matk-Schuchardt).
4. Distilled water
5. 10 ml of strong ammonia: It is prepared by weighing 6.75 gm of strong ammonia in a amber colored bottle and by adding 75 ml of strong ammonia liquid and 25 ml of distilled water to make the solution to 100 ml.
6. 5 ml of MgSO_4_: By dissolving 1.25 gm of MgSO_4_ in 100 ml of distilled water.
7. EDTA solution: 4.75 gm of EDTA powder dissolved in 250 ml of distilled water and used as a titer.
8. Calcium carbonate powder
9. Vaseline (Silicone wax)
10. Dilute HCl.

Indicators

1. Solochrome black or Mordant black

- Ca(OH)_2_ + CMCP
- Ca(OH)_2_ + propylene glycol (100%)
- Ca(OH)_2_ + distilled water.

All the three calcium hydroxide pastes were made by mixing 50 mg of Ca(OH)_2_ with 3 cc of the appropriate vehicle on the glass slab. Calcium hydroxide powder was preweighed and then stored in autoclaved vials.

Armamentarium

- Glass slab
- Cement spatula
- Glass beakers:
  - 250 ml - 6 in number
  - 100 ml - 9 in number (Borosil India Ltd, Mumbai)
  - 25 ml - 27 in number
- Test tubes - 9 in number (Borosil India Ltd, Mumbai)
- Two plastic containers
- Filter paper
- pH paper with a range of 6.5 to 9.0 (Glaxo Laboratories India Ltd, Mumbai)
- Six catheters - 07
- Glass bead stirrers - 9 in number
- Butter paper or tracing paper - 1 roll
- Modified glass test tube - 2 in number
- Stand with clamps - 6 in number.

Separate insturments (like glass slab, cement spatula) were used for the preparation of each paste under sterile conditions.

Method

The techniques used for this investigation were to determine the rate of CO_2_ absorption by Ca(OH)_2_ powder. 50 mg of Ca(OH)_2_ powder was taken in breaker and mixed with 2 cc of the vehicle (for example, CMCP or propylene glycol or distilled water).

**Fig. 5:**
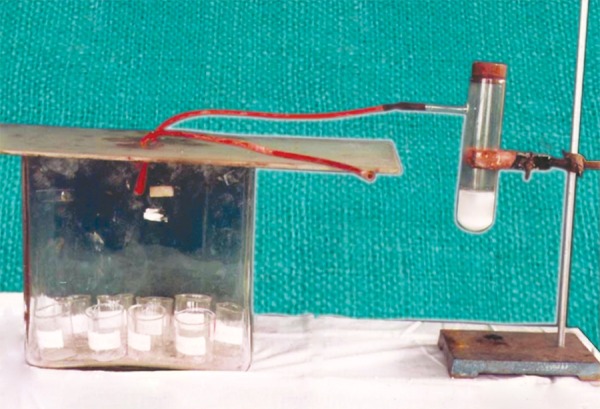
Glass jar containing 3 samples each of Ca(OH)_2_ + vehicles (H_2_O, CMCP and PG ). CO_2_ is produced in test tube

The breakers were kept inside the plastic containers, for this study 25 ml breaker were used. The holes were made on the plastic container’s lid and two catheter tubes were inserted into it. The lid of the container was closed and sealed all around with Silicone wax, and also the catheters. This was done in order to prevent the leakage of CO_2_ from the container. One of the cathater tubes was attached to a modified glass test tube with a side nozzle. The other catheter tube had no connection and was free. Calcium carbonate powder was poured into the modified glass test tube and dilute HCl was then added to it. The mouth of the modified glass test tube was closed with a cork. Carbon dioxide thus prepared was passed for 20 to 30 minutes. The specimens in the container were kept for (1 day, 7 days and 1 month). For each experiment nine (25 ml) beakers were used, 3 breakers for each specimen. The container lid was opened and the breakers were taken out (Fig. 5).

The paste was mixed with 100 ml of distilled waster and later on filtered.

pH of the filtered solutions were found out for each of the three specimens.

The final part of the experiment was calcium estimation. 50 ml of the specimen solution was taken in a conical flask. 70 ml of strong ammonia and 5 ml of MgSO_4_ were added to the solution.

Mordant black or Solochrome black indicator was used for the estimation.

The solution was titrated with EDTA solution till bluish color was obtained. A blank experiment without the sample was also performed.

## OBSERVATIONS

### Diffusibility of Ca(OH)_2_ Pastes (Tables 1 to 3)

In the diffusion rate studies of Ca(OH)_2_ with different vehicles like distilled water, propylene glycol and CMCP used to make a paste of Ca(OH)_2_, the results show a marginal difference (increase) in the pH obtained at the end of 7th, 14th and 30th days with CMCP. In all the three cases, the onset and pattern of diffusion are more or less similar. The higher pH obtained with Ca(OH)_2_ constituted in CMCP can be attributed to the protection imparted by CMCP to the already diffused Ca(OH)_2_.

### Degradation of Ca(OH)_2_ Pastes or Stability of Ca(OH)_2_ in CO_2_ Atmosphere (Tables 4 to 6)

The comparative stability of Ca(OH)_2_ constituted in the three vehicles H_2_O, propylene glycol and CMCP in an exaggerated atmosphere of CO_2_ was studied and the results show that consistently significant protection is imparted to Ca(OH)_2_ by CMCP over widely ranging periods, e.g. one day, seven days and may be responsible for the increased stability even in an increased concentration of CO_2_

## TITRATION RESULTS FOR DEGRADATION OF Ca(OH)_2_ PASTES

### One Day Results

CMCP + Ca(OH)_2_

C_1_: Out of 47.5 mg of Ca(OH)_2_, 5.9 mg is unreacted Ca(OH)_2_ solution and remaining is converted into CaCO_3_
C_2_: Out of 47.5 mg of Ca(OH)_2_, 5.9 mg is unreacted Ca(OH)_2_ solution and remaining is converted into CaCO_3_
C_3_: Out of 47.5 mg of Ca(OH)_2_, 7.4 mg is unreacted Ca(OH)_2_ solution and remaining is converted into CaCO_3_

**Table 1:** **Table 1:** Diffusibility of Ca(OH)_2_ pastes Ca(OH)_2_ + H_2_O pH values

*Samples*		*1st day*		*2nd day*		*3rd day*		*4th day*		*5th day*		*6th day*		*7th day*		*14th day*		*21st day*		*30th day*	
A_1_		7.50		7.50		8.00		8.00		8.00		8.00		8.00		8.00		8.00		8.00	
A_2_		7.00		7.50		8.00		8.00		8.00		8.00		8.00		8.00		8.00		8.00	
A_3_		7.00		7.50		8.00		8.00		8.00		8.00		8.00		8.00		8.00		8.00	
A_4_		7.00		7.50		8.00		8.00		8.00		8.00		8.00		8.00		8.00		8.50	
A_5_		7.50		7.50		7.50		8.00		8.00		8.00		8.00		8.00		8.50		8.50	
A_6_		7.00		7.00		7.50		7.50		7.50		8.00		8.00		8.00		8.00		8.00	

**Table 2:** **Table 2:** Diffusibility of Ca(OH)_2_ pastes Ca(OH)_2_ + PG pH values

*Samples*		*1st day*		*2nd day*		*3rd day*		*4th day*		*5th day*		*6th day*		*7th day*		*14th day*		*21st day*		*30th day*	
B_1_		8.00		8.00		7.50		8.00		8.00		8.00		8.00		8.00		8.00		8.00	
B_2_		7.00		8.00		8.00		8.00		8.00		8.00		8.00		8.00		8.00		8.00	
B_3_		7.50		7.50		7.50		8.00		8.00		8.00		8.00		8.00		8.00		8.00	
B_4_		8.50		8.50		8.50		8.50		8.50		8.50		8.50		8.50		8.50		8.50	
B_5_		8.00		8.00		8.00		8.00		8.00		8.00		8.00		8.00		8.00		8.00	
B_6_		8.00		8.00		8.00		8.00		8.00		8.00		8.00		8.00		8.00		8.00	

**Table 3:** **Table 3:** Diffusibility of Ca(OH)_2_ pastes Ca(OH)_2_ + CMCP pH values

*Samples*		*1st day*		*2nd day*		*3rd day*		*4th day*		*5th day*		*6th day*		*7th day*		*14th day*		*21st day*		*30th day*	
C_1_		7.50		7.50		7.50		7.50		7.50		7.50		7.50		8.00		8.50		8.50	
C_2_		7.50		7.50		7.50		7.50		7.50		8.00		8.50		8.50		8.50		8.50	
C_3_		8.00		8.00		8.00		8.00		8.00		8.00		8.50		8.50		8.50		8.50	
C_4_		8.00		8.00		8.00		8.00		8.00		8.00		8.50		8.50		8.50		8.50	
C_5_		8.50		8.50		8.50		8.50		8.50		8.50		8.50		8.50		8.50		8.50	
C_6_		9.00		9.00		9.00		9.00		9.00		9.00		9.00		9.00		9.00		9.00	

**Table 4:** **Table 4:** Degradation of Ca(OH)_2_ powder by CO_2_ Ca(OH)_2_ + PG; Ca(OH)_2_ + CMCP; Ca(OH) _2_ + H_2_O one day pH readings

*Ca(OH) _2_ + CMCP*				*Ca(OH) _2_ + PG*				*Ca(OH) _2_ + H_2_O*	
C_1_		8.79		B_1_		8.50		A_1_ 7.90	
C_2_		9.02		B_2_		8.13		A_2_ 8.01	
C_3_		8.63		B_2_		8.00		A_3_ 8.09	

**Table 5:** **Table 5:** Degradation of Ca(OH)_2_ powder by CO_2_ Ca(OH)_2_ + CMCP; Ca(OH)_2_ + PG; Ca(OH) _2_ + H_2_O seven day pH readings

*Ca(OH) _2_ + CMCP*				*Ca(OH) _2_ + PG*				*Ca(OH) _2_ + H_2_O*	
C_1_		8.50		B_1_		8.50		A_1_ 8.00	
C_2_		8.50		B_2_		8.00		A_2_ 8.00	
C_3_		8.50		B_2_		8.00		A_3_ 8.00	

**Table 6:** **Table 6:** Degradation of Ca(OH)_2_ powder by CO_2_ Ca(OH) _2_ + CMCP; Ca(OH)_2_ + PG; Ca(OH)_2_ + H_2_O one month pH readings

*Ca(OH) _2_ + CMCP*				*Ca(OH) _2_ + PG*				*Ca(OH) _2_ + H_2_O*	
C_1_		8.50		B_1_		8.00		A_1_ 9.00	
C_2_		8.50		B_2_		7.50		A_2_ 9.00	
C_3_		8.50		B_2_		8.50		A_3_ 9.00	

PG + Ca(OH)_2_

B_1_: Out of 47.5 mg of Ca(OH)_2_, 1.4 mg is unreacted Ca(OH)_2_ solution and remaining is converted into CaCO_3_.
B_2_: Out of 47.5 mg of Ca(OH)_2_, 1.4 mg is unreacted Ca(OH)_2_ solution and remaining is converted into CaCO_3_.
B_3_: Out of 47.5 mg of Ca(OH)_2_, 1.4 mg is unreacted Ca(OH)_2_ solution and remaining is converted into CaCO_3._

H_2_O + Ca(OH)_2_

A_1_: Out of 47.5 mg of Ca(OH)_2_, 1.4 mg is unreacted Ca(OH)_2_ solution and remaining is converted into CaCO_3_.
A_2_: Out of 47.5 mg of Ca(OH)_2_, 1.4 mg is unreacted Ca(OH)_2_ solution and remaining is converted into CaCO_3_.
A_3_: Out of 47.5 mg of Ca(OH)_2_, 2.2 mg is unreacted Ca(OH)_2_ solution and remaining is converted into CaCO_3_.

### One Week Results

Ca(OH)_2_ + CMCP

C_1_: Out of 47.5 mg of Ca(OH)_2_, 10.3 mg is unreacted Ca(OH)_2_ solution and remaining is converted into CaCO_3_.
C_2_: Out of 47.5 mg of Ca(OH)_2_, 10.3 mg is unreacted Ca(OH)_2_ solution and remaining is converted into CaCO_3_.
C_3_: Out of 47.5 mg of Ca(OH)_2_, 17 mg is unreacted Ca(OH)_2_ solution and remaining is converted into CaCO_3_.

Ca(OH)_2_ + PG

B_1_: Out of 47.5 mg of Ca(OH)_2_, 4.44 mg is unreacted Ca(OH)_2_ solution and remaining is converted into CaCO_3_.
B_2_: Out of 47.5 mg of Ca(OH)_2_, 2.96 mg is unreacted Ca(OH) _2_ solution and remaining is converted into CaCO_3_.
B_3_: Out of 47.5 mg of Ca(OH)_2_, 5.18 mg is unreacted Ca(OH)_2_ solution and remaining is converted into CaCO_3_.

Ca(OH)_2_ + H_2_O

A_1_: Out of 47.5 mg of Ca(OH)_2_, 4.44 mg is unreacted Ca(OH)_2_ solution and remaining is converted into CaCO_3_.
A_2_: Out of 47.5 mg of Ca(OH)_2_, 2.96 mg is unreacted Ca(OH)_2_ solution and remaining is converted into CaCO_3_.
A_3_: Out of 47.5 mg of Ca(OH)_2_, 5.18 mg is unreacted Ca(OH)_2_ solution and remaining is converted into CaCO_3_.

### One Month Results

CMCP + Ca(OH)_2_

C_1_: Out of 47.5 mg of Ca(OH)_2_, 17 mg is unreacted Ca(OH)_2_ solution and remaining is converted into CaCO_3_.
C_2_: Out of 47.5 mg of Ca(OH) _2_, 24.4 mg is unreacted Ca(OH)_2_ solution and remaining is converted into CaCO_3_.
C_3_: Out of 47.5 mg of Ca(OH)_2_, 17 mg is unreacted Ca(OH)_2_ solution and remaining is converted into CaCO_3_.

PG + Ca(OH)_2_

B_1_: Out of 47.5 mg of Ca(OH)_2_, 5.18 mg is unreacted Ca(OH)_2_ solution and remaining is converted into CaCO_3_.
B_2_: Out of 47.5 mg of Ca(OH)_2_, 4.44 mg is unreacted Ca(OH)_2_ solution and remaining is converted into CaCO_3_.
B_3_: Out of 47.5 mg of Ca(OH)_2_, 4.44 mg is unreacted Ca(OH)_2_ solution and remaining is converted into CaCO_3_.

Ca(OH)_2_ + H_2_O

A_1_: Out of 47.5 mg of Ca(OH)_2_, 6.66 mg is unreacted Ca(OH)_2_ solution and remaining is converted into CaCO_3_.
A_2_: Out of 47.5 mg of Ca(OH)_2_, 5.92 mg is unreacted Ca(OH)_2_ solution and remaining is converted into CaCO_3_.
A_3_: Out of 47.5 mg of Ca(OH)_2_, 5.92 mg is unreacted Ca(OH)_2_ solution and remaining is converted into CaCO_3_.

## DISCUSSION

The important role of Ca(OH)_2_ in dentistry, in general and endodontics in particular, has been widely recognized and it enjoys considerable popularity amongst clinicians. It finds applications in several clinical situations like direct pulp capping, indirect pulp capping, pulpotomies, weeping canals, conservative management of large apical pathologies, and also to promote apexification. Hence, any investigation aimed at explaining its action will add to the existing knowledge.

When used in the form of a paste, either distilled H_2_O or the highly tissues irritant CMCP has been commonly used as a vehicle. Both the vehicles have certain deficiencies which can adversely affect the biological qualities of Ca(OH)_2_ by directly or indirectly altering the chemical purity of Ca(OH)_2_. CMCP is known to react with Ca(OH)_2_ and yields calcium p-chlorophenolate, which is a week salt (Donald Anthony).^1,6^

Ca(OH)_2_ can undergo degradation and alteration changes, also chemical qualities due to several other causes. In both the situations, the insoluble products can interfere with the biological qualities of the material and also with the intracanal instrumentation. Hence, a biologically inert vehicle which does not suffer from these drawbacks could be though used as early as possible (1962 by Laws), namely Propylene glycol possesses some of the desirable qualities, and yet for reasons not known has not found favor among the clinicians. This alcohol is chemically inert and is also said to possess hygroscopic qualities. These two qualities offer interesting possibilities to its clinical use, when introduced into the root canal in the form of paste of Ca(OH)_2_. The hygroscopic nature of the vehicle can attract the water from the surrounding tissues, which in turn can dissolve the Ca(OH)_2_ freely and liberate it in the form of a solution to the surroundings of the root canal, and help Ca(OH)_2_ in its biological influences continuously over a long period of time. While this is a possibility, scientific literature does not indicate that such a phenomenon has been subject to scrutiny.^5,7^

Theoretically, this process can go on indefinitely as solubility of Ca(OH)_2_ solution is 1:600 though the diffusibility and degradation of Ca(OH)_2_ has got a profound influene in its clinical behavior. Literature survey has indicated that very little attention has been given by investigators to understand this aspect of the problem. Hence, the present investigation assumes relevance and considerable significance. Diffusion studies by Starhle, Hasselgren, Kazuhiko Ida, Schroder, Gordon, Prosser Zmener and Barnett to investigate this aspect have not been successful in explaining the problem adequately.^5,8^

Regarding the technique adopted for investigation of this problem, it is essential to conduct any investigation for clinical application in a clinical situation or atleast such an investigation should be conducted using extracted teeth in the laboratory. Such investigation using natural teeth are feasible, but are very few and the control of the experimental procedure in clinical situations is far more difficult. In the absence of earlier studies on these aspects, it was found necessary to conduct *in vitro* studies using the porcelain caps, which are porous in nature, and hence suitable for this preliminary investigation of the hitherto unexplained aspect of the behavior of Ca(OH)_2_. The degradation studies was conducted in an exaggerated atmosphere of 100% CO_2_, to accelerate the process of carbonation in the laboratory, which is one of the main reasons for degradation of Ca(OH)_2_.^9,10^

The assumption that Ca(OH)_2_ can undergo change only into carbonate form is not fully valid as many other chemical changes can be expected in the clinical situation. Yet, investigations to this single major factor for degradation can help the clinician in his clinical practice by indicating factors influencing the storage and use. Hence, there is a definite need for further elaborate studies on the solubility, diffusibility and degradation of calcium hydroxide. Similarly, Ca(OH)_2_ can undergo degradation in storage due to the atmospheric CO_2_. However, the studies by Cohen has revealed that such changes are minimal, and as such may not be very significant regarding the loss of activity in storage. The verification of this finding is also of clinical importance. Hence, the investigations has been taken up.

Ca(OH)_2_ form a paste with propylene glycol as a vehicle apparently diffuses on the first day itself, and the pH is maintained at around 8.0 throughout the situation of water from the surrounding medium and consequently solution of calcium hydroxide. However, this aspect needs further studies.

As evident from the result, Ca(OH)_2_ readily diffuse from a paste with CMCP as a vehicle into the surrounding medium. The rate of diffusion is similar to propylene glycol. The pH is around 8.00 from the first day and a consistently higher pH of 8.5 is attained at 14th day and is continued to 30th day. In general, this tendency for a higher pH level is exhibited by this paste. A satisfactory explanation for this phenomena is difficult without further investigation of the properties of CMCP as a vehicle. The pH of CMCP solution is 3.5 which is highly acidic. The pH of calcium hydroxide can vary between 9 to 13 depending on a variety of factors and the pH level attained by all the specimens is around 8.5, which is definitely alkaline and desirable from the biological aspect.^6,11^

The two vehicles propylene glycol and CMCP seem to be capable of making calcium hydroxide available adequately to maintain an alkaline pH from the first day itself as compared to water. The formation of crystals of calcium carbonate when water is used as a vehicle and calcium chlorophenolate when CMCP is used can be a disadvantage in root canal therapy, as they can make the removal of the interim paste filling difficult.^5,8^

Degradation studies indicate that in an exaggerated experimental condition, presence of CO_2_ in a very high proportion, all these pastes show certain degree of degradation. The pastes with water and propylene glycol appear to undergo degradation to a greater degree than that with CMCP. Even after degradation sufficient amount of calcium hydroxide is found to be left intact, which probably could be sufficient for exerting the biological properties attributed to the alkalinity. However, in actual use such an exaggerated environmental conditions are unlikely to prevail, and hence the degradation apparent in this study need not lead us to an inference that significant loss of activity can occur because of carbonation. Existence of other chemical substance in the tissue fluid which may adversely affect the activity and cannot be ruled out, can be responsible for the loss of activity of calcium hydroxide. The significance of the findings are mainly relevant to the storing conditions in chemical use. Under normal atmospheric condition, where the carbon dioxide is very limited, we can expect that all these pastes could retain their activity without undergoing degradation. Cohen has demonstrated *in vitro* Ca(OH)_2_ which was left for a long time in open, the carbonation reached as high as 30% in one month, but the pH still remained at 12.5. Similarly, when Ca(OH)_2_ is used with other vehicles in paste form, a possible degradation and loss of activity of material may take place, which in turn depends upon the ecological canal status and the factors influencing the efficacy of the medication. Even after degradation, sufficient amount of Ca(OH)_2_ is found to be left intact which probably could be sufficient for exerting the biological properties attributed to alkalinity.^1,4^

While interpreting the results of this study and assessing its significance, several aspects have to be taken into consideration. The porcelain cap method of assessing diffusibility can never be similar to the conditions prevailing in actual use when the paste remains in the root canal, which communicates with the apical zone through the apical foraman. In a clinical situation, a direct contact of the paste with diffusion through a porous material, as observed in the study, will not occur. The porous material as observed in the physical properties of a semipermeable membrane through which diffusion can occur due to phenomenon of osmosis. Hence, a diffusion study using extracted natural teeth conducted under controlled condition is warranted for better appreciation of the problem. While the degradation study has reconfirmed the earlier findings, it does not elucidate any new aspect of the problem.The more relevant study would be the assessment for degradation of chemically complex tissue fluids and the contents of the root canal and the dentinal tubules, which may have bacterial metabolic products. Such a study could add significantly to the existing knowledge. Earlier studies on these problems being limited, it is difficult to make any comparison of the present findings with those already published in the literature.^7,12^

Gordon and Prosser had demonstrated *in vitro* using extracted teeth that Ca(OH)_2_ sealed in the root canal, which can diffuse into the surrounding medium and the present study reconfirms these findings. The factor that is to be noted is the difference in the present study is the use of a porous porcelain cap across which calcium hydroxide can diffuse freely all round. Further studies are necessary to explain the phenomenon of diffusibility to find out whether diffusing from the root canal. Calcium hydroxide apparently can diffuse into the surrounding tissue across the dentine and cementum barrier in addition to diffusion into the apical area through the apical foraman. Indirect indication to such a possibility is evident from the favorable clinical reports of arrest of resorption process, when calcium hydroxide used as an intercanal therapeutic agent to control this clinical condition.^7,8^

## CONCLUSIONS

The *in vitro* studies of the diffusibility and degradation of calcium hydroxide from three different pastes prepared using water, propylene glycol and CMCP have indicated that:

1. Calcium hydroxide readily diffuse from all the three pastes.
2. There is a delay of about two days for diffusion from the paste prepared with water.
3. The final diffusion at the end of 30 days can increase the alkalinity of the surrounding medium, i.e. water to a pH of 8.5.
4. Calcium hydroxide diffuses marginally less from a paste made with propylene glycol as compared to CMCP paste.
5. Calcium hydroxide degrades by the formation of carbonates from all the pastes when stored in an atmosphere of 100% carbon dioxide.
6. Even after the degardation, sufficient calcium hydroxide is found to be left behind.
7. In view of the biologically acceptable qualities of propylene glycol and its other desirable features, it can be recommeneded for use as a vehicle for making a paste for clinical use.
8. Being a laboratory study, the findings and reconfirmation and also an investigation in an actual clinical situation is warrented for better appreciation of the qualities of the material.
